# Inbreeding shapes the evolution of marine invertebrates

**DOI:** 10.1111/evo.13951

**Published:** 2020-04-07

**Authors:** Kevin C. Olsen, Will H. Ryan, Alice A. Winn, Ellen T. Kosman, Jose A. Moscoso, Stacy A. Krueger‐Hadfield, Scott C. Burgess, David B. Carlon, Richard K. Grosberg, Susan Kalisz, Don R. Levitan

**Affiliations:** ^1^ Department of Biological Science Florida State University Tallahassee Florida 32304; ^2^ Department of Biology University of Alabama at Birmingham Birmingham Alabama 35294; ^3^ Department of Ecology and Evolution Stony Brook University Stony Brook New York 11794; ^4^ The Biology Department Bowdoin College Brunswick Maine 04011; ^5^ Schiller Coastal Studies Center Bowdoin College Orr's Island Maine 04066; ^6^ Coastal and Marine Sciences Institute University of California Davis Davis California 95616; ^7^ Department of Ecology and Evolutionary Biology University of Tennessee Knoxville Knoxville Tennessee 37996

**Keywords:** Inbreeding, marine invertebrate, mating system

## Abstract

Inbreeding is a potent evolutionary force shaping the distribution of genetic variation within and among populations of plants and animals. Yet, our understanding of the forces shaping the expression and evolution of nonrandom mating in general, and inbreeding in particular, remains remarkably incomplete. Most research on plant mating systems focuses on self‐fertilization and its consequences for automatic selection, inbreeding depression, purging, and reproductive assurance, whereas studies of animal mating systems have often assumed that inbreeding is rare, and that natural selection favors traits that promote outbreeding. Given that many sessile and sedentary marine invertebrates and marine macroalgae share key life history features with seed plants (e.g., low mobility, modular construction, and the release of gametes into the environment), their mating systems may be similar. Here, we show that published estimates of inbreeding coefficients (*F*_IS_) for sessile and sedentary marine organisms are similar and at least as high as noted in terrestrial seed plants. We also found that variation in *F*_IS_ within invertebrates is related to the potential to self‐fertilize, disperse, and choose mates. The similarity of *F*_IS_ for these organismal groups suggests that inbreeding could play a larger role in the evolution of sessile and sedentary marine organisms than is currently recognized. Specifically, associations between traits of marine invertebrates and *F*_IS_ suggest that inbreeding could drive evolutionary transitions between hermaphroditism and separate sexes, direct development and multiphasic life cycles, and external and internal fertilization.

Through its effects on homozygosity, effective population size, and recombination rates, the extent to which individuals inbreed or outbreed strongly influences the distribution and maintenance of genetic variation among individuals and populations (Charlesworth 2003). Historically, studies of terrestrial plant mating systems have emphasized rates of self‐fertilization with the associated benefits of reproductive assurance and the genetic transmission advantage relative to the costs of inbreeding depression (Goodwillie et al. 2005). In contrast, studies of terrestrial animal mating systems have generally focused on variance in mate number (Emlen and Oring 1977; Arnold 1994), and often assumed that behavioral adaptations limit inbreeding (Pusey and Wolf 1996). Because of the severe inbreeding depression manifested in species with outbreeding evolutionary histories (Keller and Waller 2002), the adaptive value of inbreeding is often not considered in animals (Duthie and Reid 2016). More recently, however, genetic evidence for widespread inbreeding in natural populations of a diverse array of animals has fueled renewed interest in the distribution of mating systems across taxa, and the evolutionary, behavioral, ecological, and genetic causes and consequences of deviations from panmixia (Jarne and Auld 2006; Kokko and Ots 2006; Puurtinen 2011; Szulkin et al. 2013).

Compared to terrestrial ecosystems, the marine environment has long been considered to be demographically open, such that the lack of obvious barriers to gene flow, presence of planktonic developmental stages, or both promote dispersal and decrease the possibility of inbreeding (Strathmann 1990; Knowlton and Jackson 1993; Caley et al. 1996). Nevertheless, over the last several decades indirect and direct evidence has surfaced indicating that fine scale population structure and inbreeding occurs in at least some marine invertebrates (Carlon 1999; Addison and Hart 2005; Hellberg 2009) and macroalgae (Valero et al. 2001; Krueger‐Hadfield and Hoban 2016).

Whether these findings are artifacts of the methods used to detect inbreeding (e.g., the prevalence of null alleles that inflates estimates of homozygosity; Addison and Hart 2005) or undetected spatial structure (i.e., Wahlund effects), or whether they truly reflect deviations from panmixia within populations, remains an open question (Addison and Hart 2005; Waples 2015, 2018). However, several lines of evidence suggest that observations of inbreeding in marine organisms may represent a more central component of their biology than generally assumed (Knowlton and Jackson 1993). First, many benthic marine invertebrates have sessile or sedentary adult stages, restricting opportunities for gene flow primarily to planktonic propagules, such as sperm, eggs, and larvae. Second, the distances over which sperm in particular remains viable and at sufficiently high density for effective fertilization may be on the scale of meters, or less (Grosberg 1991; Manriquez et al. 2001; Levitan 2002; but see Yund et al. 2007). Third, numerous species entirely lack planktonic dispersal, or have brief larval durations (Shanks 2009). Even in species with prolonged planktonic phases, offspring can return to natal patches (Christie et al. 2010; Burgess et al. 2014) or remain associated with siblings through collective dispersal (Eldon et al. 2016). Consequently, as in many terrestrial angiosperms, the interplay between inbreeding and outbreeding could be a critical element to the evolution of mating systems in marine organisms (Knowlton and Jackson 1993; Carlon 1999; Kamel and Grosberg 2013).

On the other hand, differences in the physical properties of air and seawater, and therefore in the way that gametes, larvae, and seeds disperse in terrestrial and marine environments, could drive important discrepancies in mating systems and the prevalence of inbreeding (Strathmann 1990; Denny 1993; Knowlton and Jackson 1993). For example, pollen is often transferred by animal vectors in terrestrial systems, and fluctuations in pollinator abundance can determine selfing rates in terrestrial seed plants (Lloyd 1992). Pollinators, however, are exceptionally rare in aquatic systems (Strathmann 1990; but see van Tussenbroek et al. 2016), with gamete transfer primarily occurring via water currents, diffusion, or copulatory structures in marine organisms (Strathmann 1990; Vermeij and Grosberg 2017). Because distributions of outcrossing rates vary in plants with biotic or wind pollination (Goodwillie et al. 2005), differences in animal‐assisted versus passive gamete transfer may contribute to disparities in the mating systems of terrestrial and marine organisms. Furthermore, phylogenetic variation in the magnitude and/or genetic architecture of inbreeding depression has the potential to influence the distribution of inbreeding among animals, plants, and macroalgae (Charlesworth and Charlesworth 1999).

Relationships among life history traits, the magnitude of inbreeding, and patterns of genetic diversity have been well established in terrestrial plants (Loveless and Hamrick 1984). Differences in the capacity for self‐fertilization and in pollen and seed transport mechanisms are known to have important effects on mating systems (Hamrick and Godt 1996). Similar to floral morphology, the arrangement of sexual organs varies among species of marine animals, influencing the ability to self‐fertilize (Jarne and Auld 2006), and may contribute to variation in the prevalence of inbreeding. Mating systems also differ among plant species with seeds dispersed by wind, animal vectors, or gravity, and this variation in the potential for dispersal is mirrored in marine propagules with different planktonic durations. Whether these features that affect the expression of inbreeding in land plants are also at work in marine organisms is important to determining if terrestrial and marine systems are fundamentally unique (Webb 2012).

Overall, there is a presumption that because self‐fertilization is common in terrestrial seed plants (Whitehead et al. 2018), and because inbreeding depression is often severe in animals (Keller and Waller 2002), inbreeding is relatively rare in marine invertebrates. This notion is exemplified in previous reviews which state, “Inbreeding is more extreme in plants than in sessile marine invertebrates…although the scarcity of data makes it difficult to estimate the frequency with any accuracy” (Knowlton and Jackson 1993, p. 247), or conclude that evidence of high levels of homozygosity “does not imply that marine invertebrates with planktonic sperm are typically inbred (via mating with relatives)” (Addison and Hart 2005, 452). This context suggests that a quantitative assessment of inbreeding in marine and terrestrial organisms, as well as an assessment of variation in inbreeding within marine invertebrates, is warranted.

To explore similarities and discrepancies in the prevalence of inbreeding in the mating systems of terrestrial seed plants, marine macroalgae, and marine invertebrates, we evaluated patterns in published estimates of the inbreeding coefficient (*F*_IS_). *F*_IS_ integrates the effect of inbreeding on homozygosity by quantifying the deviation of observed genotype frequencies from those expected under Hardy‐Weinberg equilibrium (Charlesworth 2003). Inbreeding, null alleles, and undetected genetic structure increase observed homozygosity and *F*_IS_, whereas outbreeding, mutation, and inbreeding depression tend to reduce *F*_IS_ (Stoeckel et al. 2006; Waples 2018). We compared *F*_IS_ in terrestrial seed plants, marine macroalgae, and marine invertebrates, and examined how the potential for dispersal, mate choice, and self‐fertilization influences levels of inbreeding within marine invertebrates. Given the prevalence of self‐fertilization in terrestrial seed plants (Whitehead et al. 2018), and the presumption that inbreeding avoidance is commonplace in most animal mating systems (Pusey and Wolf 1996), we, like others (Knowlton and Jackson 1993), expected to find elevated *F*_IS_ in terrestrial seed plants compared with marine organisms. Instead, we found that the degree of inbreeding in sessile and sedentary marine organisms is at least as great as noted for terrestrial seed plants. Furthermore, variation in inbreeding within marine invertebrates could be explained by patterns of dispersal, the degree of control over matings, and the ability to self‐fertilize. Overall our results suggest that the lifestyle commonalities of plants, macroalgae, and marine invertebrates including a sedentary adult phase and the use of external vectors to transport gametes and propagules may shape similar mating systems despite large divergences in the physical properties of air and water, and phylogenetic history.

## Methods

### INBREEDING COEFFICIENTS

We assembled data on inbreeding coefficients (*F*_IS_) for 142 species of terrestrial seed plants, 200 species of marine invertebrates, and 41 species of marine macroalgae. We chose these groups because they share traits associated with a sessile or sedentary lifestyle, with dispersal primarily via gametes or propagules, and because they span marine and terrestrial environments.

Inbreeding coefficients were obtained from the primary literature, either from our own systematic searches or from previous surveys. Articles were included in the study if they reported either *F*_IS_ or expected (*H*_E_) and observed (*H*_O_) heterozygosities from which the inbreeding coefficient could be calculated using the formula $F_{\mathrm{IS}}=\;\frac{H_{E}-\;H_{O}}{H_{E}}$. We used a database of inbreeding coefficients for terrestrial seed plants compiled by the National Evolutionary Synthesis Center (NESCent) workshop “Understanding the paradox of mixed mating in flowering plants.” The NESCent database contains *F*_IS_ and outcrossing rates for 150 species of seed plants often averaged over multiple populations and was previously used to assess factors contributing to the prevalence of mixed mating (Goodwillie et al. 2005). We removed marine and aquatic species and evaluated *F*_IS_ in 142 species of terrestrial seed plants.

For the invertebrates, we started with the 97 species examined in 83 studies cited in the review by Addison and Hart (2005) and returned to the original papers to obtain population‐level data. Mating systems are known to vary considerably among populations of terrestrial seed plants, and we wanted to incorporate this source of variation in our analysis of marine invertebrates. For each species, we collected population‐level estimates of *F*_IS_ when available or we recorded the single overall estimate of *F*_IS_ when this was all that was reported. We expanded this survey by searching in Web of Science for articles published between 2005 and 2017 with the key words “population structure,” “population genetics,” and “marine invertebrate.” We also surveyed the journals *Molecular Ecology*, *Marine Ecology Progress Series*, and *Marine Biology* for articles published during this period that contained inbreeding coefficients for marine invertebrates. We added 104 additional studies with 103 species for a total of 2036 population estimates in 200 unique species spanning 10 phyla.

Data on marine macroalgae were obtained using the search engine Scopus with the key words “alga” and either “mating system” or “microsatellite.” The macroalgae, or seaweeds, are a polyphyletic group of organisms occupying the intertidal and subtidal littoral zone around the world. We included marine macroalgae from the Ochrophytes (brown macroalgae; Laminariales, Fucales, Tilopteridales, and Ectocarpales), the Rhodophytes (red macroalgae; Gigartinales, Halymeniales, Gracilariales, Corallinales, and Gelidiales), and the Chlorophytes (green macroalgae; Ulvales, Cladophorales) in our study, united by their functional similarities in the habitats in which they occupy as well as shared life cycle variation and complexity.

To compare levels of inbreeding across organismal groups, we averaged population‐level estimates of the inbreeding coefficient for each species of marine invertebrate and marine macroalga. Because the NESCent database contains *F*_IS_ derived exclusively from allozyme and microsatellite markers, entries for marine invertebrates and marine macroalgae that were conducted using single‐nucleotide polymorphisms (SNPs) were removed from these analyses to achieve a balanced design. Eight studies of marine invertebrate species reported *F*_IS_ estimates from both microsatellites and allozymes, so we conducted separate analyses that included these species with either their microsatellite or allozyme estimates. The adjusted dataset contained all 142 species of terrestrial seed plants, 41 species of marine macroalgae, and 180 marine invertebrate species.

### TRAIT AND MARKER CHARACTERIZATION

We evaluated the effects of different sexual, sperm transfer, and developmental traits on inbreeding coefficients exclusively within marine invertebrates. Patterns of genetic diversity associated with trait variation have already been reviewed in terrestrial seed plants (Hamrick and Godt 1996), and we found few estimates of inbreeding coefficients for marine macroalgae. For each entry, we recorded taxonomic information down to species, the type of genetic marker employed, and aspects of the organisms’ reproductive and developmental biology.

We obtained trait information for each species either from the original article reporting the inbreeding coefficient or from additional searches in the primary literature. For certain entries, it was unclear whether the organism of interest represented a distinct species or was conspecific with others in the database. In these cases, we relied on the article reporting the inbreeding coefficient and the author's assessment to classify these organisms as either the same or distinct species.

We characterized reproductive modes based on the potential for self‐fertilization and distinguished organisms with sexual configurations that at least permit the capacity to self‐fertilize from those that lack this potential. Specifically, we distinguished gonochoristic or dioecious species with separate sexes (e.g., the green sea urchin, *Strongylocentrotus droebachiensis*) from hermaphroditic species. Within hermaphrodites, we further delineated the potential for self‐fertilization by distinguishing sequentially hermaphroditic species without the ability to self (individuals transition between sexes without overlap, e.g., the ascidian *Botryllus schlosseri*) from simultaneously hermaphroditic (e.g., the coral *Orbicella annularis*) species. For some species known to be hermaphroditic, the timing of male and female sexual expression was not reported (e.g., the sponge, *Paraleucilla magna*), and we consequently characterized them as “unassigned hermaphrodites.”

We categorized sperm transfer modes to evaluate how varying degrees of adult control over mate choice contribute to inbreeding and outbreeding. In general, marine invertebrates can fertilize the eggs of conspecifics in one of three ways: (1) broadcast spawning in which eggs and sperm are released into the water column for external fertilization (e.g., the coral *Orbicella annularis*), (2) spermcasting in which sperm are released into the water column, taken up by a mate, and fertilization occurs internally (e.g., the ascidian *Botryllus schlosseri*), or (3) copulating, in which sperm are directly transferred between mates and fertilization occurs internally (e.g., the barnacle *Catomerus polymerus*). Species that are known to exhibit variation in sperm transfer mode (e.g., members of the coral genus *Pocillopora*) were excluded from the study.

Although these sperm transfer categories represent generalities, they contain within them variation on these broad themes. For example, the seastar *Parvulastra exigua* reproduces by depositing benthic egg masses and directly transferring sperm onto them (Byrne 1992). Because fertilization occurs externally in this species, we characterized this as a form of broadcast spawning with direct development. Because fertilization occurs externally during broadcast spawning, adults of these species are limited in their ability to discern between potential mates once gametes are released (Levitan 2018). Spermcasting allows for the sperm recipient to potentially choose among available mates, but individuals releasing sperm have limited control over mate choice after gamete release (Bishop and Pemberton 2006). Copulation permits the greatest degree of control over mate choice as individuals have the ability to discern between potential mates (Christy 1983).

We classified developmental modes to assess how differences in the potential for dispersal affect the degree to which organisms inbreed. We categorized marine invertebrate development as either (1) direct development with no planktonic larval stage (e.g., the sea anemone *Epiactis prolifera*), (2) lecithotrophic larval development, in which offspring rely on a limited maternally derived energy source prior to metamorphosis (e.g., the bryozoan *Bugula stolonifera*), or (3) planktotrophic larval development, in which offspring must feed in the plankton (e.g., the crown of thorns *Acanthaster planci*). Species that could not be placed into these categories, such as poecilogenous species with multiple larval developmental modes, were excluded from the study.

Species with direct development lack a planktonic stage and consequently have limited potential for dispersal. Lecithotrophic larvae spend minutes to days in the plankton and have intermediate dispersal potential. Planktotrophic larvae require weeks to months to reach competency for settlement and have the greatest dispersal potential. Although these developmental categories reflect broad differences in the potential to disperse away from relatives, there are numerous exceptions in which realized dispersal deviates substantially from that expected based on developmental mode alone (Hellberg 2009).

We also recorded the type of genetic marker employed to investigate how technical errors such as null alleles may have influenced our results. Whenever possible, we compared allozymes, microsatellites, and SNPs evaluated by sequencing. Null alleles are allelic variants that are not detected by electrophoresis, or by Sanger or next‐generation sequencing platforms. They tend to inflate *F*_IS_ from population samples because only the nonnull allele is detected in heterozygous individuals, resulting in erroneous classification as a homozygous genotype, and a consequent decrease in the reported frequency of heterozygotes.

### STATISTICAL ANALYSES

Our analyses focused on contrasting distributions of the inbreeding coefficient for terrestrial seed plants, marine invertebrates, and marine algae, and on evaluating patterns of *F*_IS_ associated with different sexual, sperm transfer, and developmental modes within marine invertebrates. We chose not to make phylogenetic adjustments in our analyses, as *F*_IS_ is reflective of evolutionarily recent inbreeding and heterozygosity returns to Hardy‐Weinberg equilibrium following a single generation of random mating (Charlesworth 2003). For some tests, data were limited or absent for a particular level of a predictor variable, and in these cases, we removed the missing level to promote a complete design matrix. All statistical analyses were conducted in the R version 3.5.1 (R Core Team 2018).

We compared distributions of species mean *F*_IS_ for each organismal group with two‐sample Kolmogorov‐Smirnov tests. Species mean *F*_IS_ could not be transformed to meet the assumptions of parametric testing and was also evaluated using a two‐factor nonparametric test with organismal group (terrestrial plants, marine macroalgae, or marine invertebrates) and marker type (allozyme or microsatellite) as predictor variables in the R package “Rfit” (Kloke and McKean 2012). We incorporated marker type in our analyses in an effort to remove variance in *F*_IS_ that could be attributed to marker‐specific errors rather than inbreeding. We explored a significant interaction between organism and marker type by conducting single factor nonparametric Kruskal‐Wallis tests among marker types within each organismal group and among organismal groups within each marker type. Because our study focused on contrasting levels of inbreeding across organismal groups, we also repeated these analyses on *F*_IS_ values that were equal to or greater than zero to remove the effects of heterozygote excess.

We evaluated the effects of different sexual, sperm transfer, and developmental modes on *F*_IS_ values for marine invertebrates with a linear mixed‐effects model in the R package “lme4” (Bates et al. 2015). We included population identity for each species and removed species for which we had fewer than three population estimates. We treated species identity as a random effect and evaluated variation in *F*_IS_ for 148 species of marine invertebrates from 1971 populations with developmental mode (direct, lecithotrophic, or planktotrophic), sperm transfer mode (broadcast spawning, spermcasting, or copulating), sexual mode (gonochoristic, sequentially hermaphroditic, simultaneously hermaphroditic, hermaphroditic but unknown sexual timing), and marker type (allozymes, microsatellites, or SNPs) as fixed effects. Sufficient data were not available for a complete design matrix that incorporated all possible interactions across all fixed effects. We consequently chose a statistical model that evaluated an interaction of interest between sperm transfer and developmental mode and accounted for differences in sexual mode and marker type as main effects. Results from alternative models with the interaction term between different combinations of these independent variables did not qualitatively change the findings of our study. We investigated significant main effects and an interaction by conducting multiple comparisons in the R package “emmeans” and adjusted for multiple comparisons with the Tukey's method (Lenth et al. 2018).

## Results

**Figure 1:**
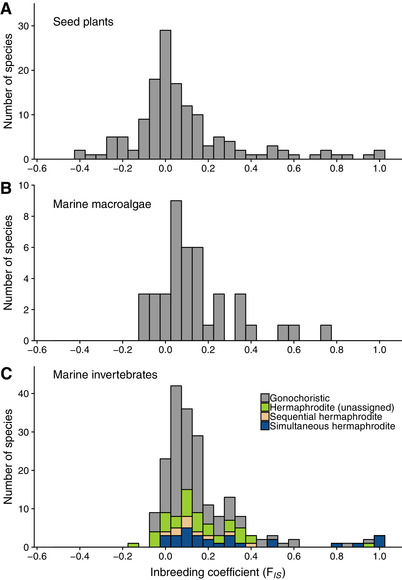
Distributions of mean *F*_IS_ per species in (A) 142 species of terrestrial seed plants, (B) 41 species of marine macroalgae, and (C) 180 species of marine invertebrates.

We compared degrees of inbreeding across terrestrial seed plants, marine macroalgae, and marine invertebrates both by analyzing the distributions of species mean *F*_IS_ for each of these broad organismal groups and by comparing the means and variances of these distributions. Here, we report results from analyses using allozyme estimates in eight marine invertebrate species with *F*_IS_ from both marker types; results using their corresponding microsatellite estimates are similar and can be found in Table S1. The distribution of *F*_IS_ in terrestrial seed plants was shifted toward lower values compared to marine macroalgae (KS test, *D* = 0.337, *P* = 0.002) and marine invertebrates (KS test, *D* = 0.343, *P* < 0.001), whereas the distributions of *F*_IS_ for macroalgae and invertebrates did not differ (KS test, *D* = 0.121, *P* = 0.715; Fig. 1). In the comparison of mean *F*_IS_ for each organismal group, we found a significant interaction between organismal group and marker type (Table 1; Fig. S1): notably, among seed plants, mean *F*_IS_ values were lower when assayed with allozymes compared to microsatellites (KW test, *χ* ^2^ = 6.787, DF = 1, *P* = 0.009). There was no difference between marker types within macroalgae (KW test, *χ* ^2^ = 0.059, DF = 1, *P* = 0.809) or invertebrates (KW test, *χ* ^2^ = 0.001, DF = 1, *P* = 0.992). Inbreeding coefficients estimated with microsatellites did not differ among taxa (KW test, *χ* ^2^ = 0.433, DF = 2, *P* = 0.805), whereas *F*_IS_ values estimated with allozymes were significantly lower in seed plants compared to marine invertebrates (KW test, *χ* ^2^ = 20.85, DF = 1, *P* < 0.001). When considering only inbreeding coefficients equal to or greater than zero, there was no difference in the distribution of *F*_IS_ in terrestrial seed plants compared to marine macroalgae (KS test, *D* = 0.241, *P* = 0.113) or marine invertebrates (KS test, *D* = 0.153, *P* = 0.134). We also found no significant differences in *F*_IS_ among organismal groups or marker types in the nonparametric ANOVA when only estimates equal to or greater than zero were considered (Table 1).

**Table 1:** Results of robust nonparametric ANOVA for the effects of organismal group (terrestrial plants, marine macroalgae, and marine invertebrates), marker type (allozyme and microsatellite), and their interaction on species level estimates of *F*_IS_. Analysis conducted separately using allozyme estimates for eight marine invertebrate species with *F*_IS_ from both marker types (first value), and using *F*_IS_ values ≥ 0 (second value). See text for details. Significant *P*‐values in bold

Source	DF	RD	Mean RD	*F*	*P*‐value
Taxon	2	0.35 | 0.12	0.18 | 0.06	2.68 | 1.19	0.070 | 0.307
Marker	1	0.08 | 0.09	0.08 | 0.09	1.16 | 1.71	0.282 | 0.192
Taxon × Marker	2	0.42 | 0.09	0.21 | 0.04	3.19 | 0.83	**0.042** | 0.438

The analysis of the effects of sexual, developmental, and sperm transfer modes on *F*_IS_ values within marine invertebrates revealed significant main effects of sexual mode and marker type, and a significant interaction between developmental and sperm transfer modes (Table 2). Species with the potential for self‐fertilization via simultaneous hermaphroditism had significantly greater *F*_IS_ than gonochoristic species and unassigned hermaphrodites (Table 3). Furthermore, estimates of *F*_IS_ were greater for microsatellites compared to allozymes, but neither microsatellite nor allozyme estimates were significantly different from those based on SNPs (Table 4).

**Table 2:** Results of linear mixed effects Type III ANOVA for effects of sperm transfer mode, developmental mode, sexual mode, marker type, and the interaction between sperm transfer mode and developmental mode on population‐level *F*_IS_. Species identity was included as a random effect. Significant *P*‐values in bold

Source	Sum of squares	Mean square	DF	*F*	*P*‐value
Sperm transfer mode	0.058	0.029	(2, 129)	2.536	0.083
Developmental mode	0.049	0.025	(2, 130)	2.164	0.119
Sexual mode	0.167	0.056	(3, 127)	4.890	**0.003**
Marker	0.137	0.069	(2, 1770)	6.029	**0.003**
Sperm transfer × Development	0.118	0.029	(4, 130)	2.582	**0.040**

**Table 3:** Results of least‐squares means comparisons of population level *F*_IS_ for sexual modes (gonochoristic [G], unassigned hermaphroditic [H], sequentially hermaphroditic [Q], and simultaneously hermaphroditic [T]) of marine invertebrates. Species identity was included as a random effect. Bold *P*‐values are significant after adjusting for multiple comparisons with Tukey's method

Comparison	Estimate	SE	df	*t* ratio	Adjusted *P*‐value
G‐H	−0.026	0.043	142	−0.611	0.928
G‐Q	−0.037	0.069	139	−0.532	0.951
G‐T	−0.187	0.049	144	−3.795	**0.001**
H‐Q	−0.010	0.076	139	−0.138	0.999
H‐T	−0.160	0.058	143	−2.758	**0.033**
Q‐T	−0.150	0.079	140	−1.891	0.236

**Table 4:** Results of least‐squares means comparisons of population‐level *F*_IS_ for marker types (allozymes [A], microsatellites [M], and SNPs [S]) within marine invertebrates. Species identity was included as a random effect. Bold *P*‐values are significant after adjusting for multiple comparisons with Tukey's method

Comparison	Estimate	SE	df	*t* ratio	Adjusted *P*‐value
A‐M	−0.060	0.015	1814	−3.971	**0.001**
A‐S	−0.035	0.021	1930	−1.625	0.235
M‐S	0.025	0.019	1952	1.312	0.389

The interaction between developmental and sperm transfer modes was apparent in two ways. Inbreeding coefficients did not differ significantly for developmental modes in species that mate by broadcast spawning or copulation; however, within spermcasting species, those with direct development had significantly higher *F*_IS_ values than lecithotrophs or planktotrophs (Table 5). Furthermore, estimates of *F*_IS_ did not vary significantly among sperm transfer modes in species with lecithotrophic or planktotrophic dispersal, but within species with direct development, spermcasters had significantly greater inbreeding coefficients than species that copulate (Table 6).

**Table 5:** Results of least‐squares means comparisons of population‐level *F*_IS_ for development modes (direct development [D], lecithotrophic [L], and planktotrophic [P]) within sperm transfer modes of marine invertebrates. Species identity was included as a random effect. Bold *P*‐values are significant after adjusting for multiple comparisons with Tukey's method

Mode	Comparison	Estimate	SE	df	*t* ratio	Adjusted *P*‐value
Broadcast spawning	D‐L	−0.009	0.184	136	−0.048	1.000
	D‐P	−0.012	0.183	136	−0.066	1.000
	L‐P	−0.003	0.044	137	−0.072	1.000
Spermcasting	D‐L	0.276	0.066	145	4.173	**0.002**
	D‐P	0.454	0.141	142	3.231	**0.040**
	L‐P	0.179	0.134	141	1.333	0.920
Copulating	D‐L	0.048	0.142	145	0.340	1.000
	D‐P	−0.014	0.066	142	−0.215	1.000
	L‐P	−0.062	0.135	145	−0.461	0.999

**Table 6:** Results of least‐squares means comparisons of population‐level *F*_IS_ for sperm transfer modes (broadcast spawning [B], spermcasting [S], and copulating [C]) within developmental modes of marine invertebrates. Species identity was included as a random effect. Bold *P*‐values are significant after adjusting for multiple comparisons with Tukey's method

Developmental mode	Comparison	Estimate	SE	df	*t* ratio	Adjusted *P*‐value
Direct	B‐S	−0.370	0.189	137	−1.954	0.578
	B‐C	0.052	0.189	137	0.273	1.000
	S‐C	0.422	0.079	145	5.371	**<0.001**
Lecithotrophic	B‐S	−0.085	0.048	137	−1.780	0.695
	B‐C	0.109	0.134	145	0.810	0.996
	S‐C	0.194	0.135	145	1.436	0.882
Planktotrophic	B‐S	0.097	0.133	141	0.727	0.998
	B‐C	0.050	0.046	138	1.072	0.977
	S‐C	0.047	0.134	141	−0.350	1.000

## Discussion

### COMPARISON ACROSS LARGE‐SCALE ORGANISMAL GROUPS

Despite dramatic differences in their evolutionary histories and in the physical properties of the media they inhabit, the distributions of positive *F*_IS_ values for 180 marine invertebrate species, 142 species of terrestrial seed plants, and 41 marine macroalgae were strikingly similar (Fig. 1). Estimates of *F*_IS_ exceeded 0.30 for 13% of seed plant species, 16% of marine invertebrates, and 17% of marine macroalgae. Although mean *F*_IS_ estimated with microsatellites did not differ among these three organismal groups, the distribution was shifted toward lower *F*_IS_ values in terrestrial seed plants compared with marine macroalgae and marine invertebrates when estimated with allozymes (Fig. S1). This shift in seed plants was associated with a higher frequency of negative *F*_IS_ values, potentially due to clonal propagation or inbreeding depression promoting heterozygote excess in some species. Nonetheless, analyses of *F*_IS_ values equal to or greater than zero found no differences in the distribution or magnitude of inbreeding in either marker type across organismal groups. Considering that differences were inconsistent among marker types and not apparent in the positive range of *F*_IS_, we conservatively conclude that inbreeding is at least as prevalent in sessile and sedentary marine organisms as it is in terrestrial seed plants.

We found a greater frequency of negative *F*_IS_ at allozyme loci in terrestrial seed plants compared with marine macroalgae and marine invertebrates (Fig. S1). The majority of these negative estimates were reported by studies that systematically contrasted *F*_IS_ in seedlings and maternal plants in an effort to estimate inbreeding depression across life stages (e.g., Yeh et al. 1983; Husband and Schemske 1995; Fady and Westfall 1997). Inbreeding depression represents selection against inbred individuals and can promote excess heterozygosity at loci influencing fitness (Charlesworth and Charlesworth 1999). Interestingly, we found that across all organismal groups, 32% of *F*_IS_ values estimated with allozyme loci were negative, whereas only 9% of *F*_IS_ values estimated with neutral microsatellite loci were negative. The greater heterozygote excess observed in allozymes may reflect differences in the manifestation of inbreeding depression at neutral compared to enzyme‐regulating loci, as has previously been reported in heterozygosity fitness correlations (Borrell et al. 2004). Similarly, negative *F*_IS_ can be a product of mutation in clonal organisms, which inflates heterozygosity relative to expectations from Hardy‐Weinberg equilibrium (Stoeckel et al. 2006).

Although estimates of *F*_IS_ can be biased upwardly by errors in genotyping (e.g., null alleles) and undetected population structure (e.g., Wahlund effect), or negatively by inbreeding depression and clonal mutation, these factors are unlikely to be a complete explanation for the patterns we and others have reported (Addison and Hart 2005). For example, *F*_IS_ for seed plants (Goodwillie et al. 2005), red macroalgae (Krueger‐Hadfield et al. 2015), and reef corals (Sherman 2008; Carlon and Lippe 2011) is correlated with measures of inbreeding from progeny arrays, suggesting that *F*_IS_ can reliably reflect recent inbreeding. Moreover, although different genetic markers have varying degrees of susceptibility to bias, in this study, they all showed the same pattern in terms of traits that influence the potential for inbreeding (Fig. S2).

Similarity in the distributions of *F*_IS_ across major organismal groups inhabiting dramatically different environments suggests that inbreeding is an influential evolutionary force in marine invertebrates, just as it is in plants and macroalgae. Furthermore, although the absolute value of *F*_IS_ may be influenced by factors other than inbreeding, including null alleles, Wahlund effects, inbreeding depression, or clonal mutation, there is no obvious reason why these potential biases would affect marine invertebrates or marine macroalgae more than plants, or differentially affect groups of marine invertebrates that differ in traits likely to influence inbreeding.

### COMPARISONS ACROSS REPRODUCTIVE AND DEVELOPMENTAL TRAITS WITHIN MARINE INVERTEBRATES

Sessile and sedentary marine invertebrates have general reproductive and dispersal features that make them likely to engage in inbreeding (Knowlton and Jackson 1993). High mortality during early life stages and large variation in reproductive success among individuals is thought to contribute to reduced effective population sizes and genetic differentiation at smaller than expected spatial scales (Hedgecock 1994). This “sweepstakes reproduction” combined with the local retention or collective dispersal of propagules may reduce genetic mixing and place relatives together in breeding units (Eldon et al. 2016). Within these general processes, particular traits that effect the movement of gametes and offspring may compound or obstruct patterns of inbreeding in marine invertebrates as they do in terrestrial seed plants.

**Figure 2:**
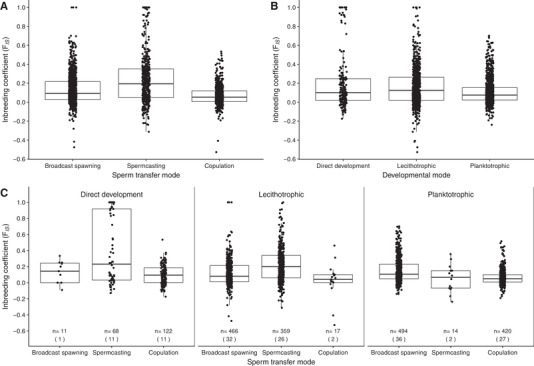
Population‐level *F*_IS_ for 148 species of marine invertebrates in relation to (A) sperm transfer mode, (B) developmental mode, and (C) both sperm transfer and developmental modes; *n* is the number of populations with number of species in parentheses.

Among 1971 populations of 148 species across 10 phyla, we detected greater *F*_IS_ in simultaneous hermaphrodites compared to species with separate sexes and a significant interaction between sperm transfer and developmental modes (Figs. 1 and 2). As expected, *F*_IS_ was greater for species with the capacity to self‐fertilize than for gonochoristic (or dioecious) species. Self‐fertilization has been described for some marine invertebrates, and available data support similar distributions of selfing rates for hermaphroditic animal taxa and terrestrial seed plants (Jarne and Auld 2006). Inbreeding coefficients were smaller for species with separate sexes than hermaphrodites (Table 3), but the range in *F*_IS_ was similar in species with separate sexes compared to species with the capacity to self‐fertilize (Fig. 1). This suggests a large contribution of biparental inbreeding to elevated *F*_IS_ in marine invertebrates.

**Figure 3:**
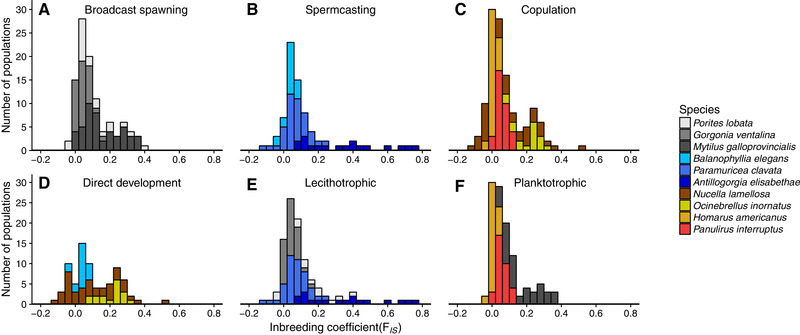
Distributions of population‐level *F*_IS_ for gonochoristic marine invertebrates with different sperm transfer (A, B, and C) and developmental modes (D, E, and F). The three species with the greatest number of population‐level estimates were selected for each sperm transfer and developmental mode.

Of the 10 separate‐sex species with the greatest number of population estimates in our dataset, seven of them had *F*_IS_ values that spanned an order of magnitude (Fig. 3). For example, *F*_IS_ in the gonochoric soft coral *Antillogorgia elisabethae* ranged from 0.06, indicating little coancestry between mates, to 0.74, signifying high levels of biparental inbreeding. Models suggest that biparental inbreeding has evolutionary dynamics comparable to those of self‐fertilization (Duthie and Reid 2016), and high interpopulation variance in *F*_IS_ of marine invertebrates might reflect diverging mating systems, as has been proposed for seed plants (Whitehead et al. 2018).

We also found a complex relationship between the effects of sperm transfer and developmental modes on *F*_IS_ (Fig. 2; Tables 5 and 6). First, in species with direct development, *F*_IS_ was significantly higher in spermcasters compared to copulators. Copulation provides adults with the greatest degree of mate choice, and given the opportunity, many species might actively avoid consanguineous mating owing to the fitness consequences of inbreeding depression (Charlesworth and Charlesworth 1987). Addison and Hart (2005) suggested that lower *F*_IS_ in copulating versus sperm‐releasing marine invertebrates was driven by unidentified factors related to the evolutionary transition from external to internal fertilization. We propose that inbreeding and the magnitude of inbreeding depression might directly influence the evolution of sperm transfer modes via the degree of control over mate choice. When eggs and sperm are released into the water for external fertilization, adults have little control over which sperm fertilize which eggs. Although the eggs of at least some species have gamete recognition systems that allow them to distinguish among different conspecific sperm (Palumbi 1999; Levitan et al. 2019), they can only exert choice when sperm from multiple males simultaneously collide with eggs during a brief window between sperm contact and fertilization (Levitan 2018). In spermcasters, females can collect and store sperm, and so may have greater opportunity to choose among more or less related males (Bishop et al. 1996). Copulation is the only mating strategy in which male and female adults can directly choose mates with high fidelity. Even when offspring do not disperse, adults may be selected to avoid mating with kin because of severe inbreeding depression. In contrast, copulating terrestrial and aquatic mollusks often have high selfing rates (Jarne and Auld 2006), which evolve in environments where limited mate availability outweighs selection against inbred offspring and favors selfing to promote reproductive assurance. The evolutionary transition from broadcast spawning to spermcasting and finally copulation might have been driven by a combination of selection for reproductive assurance and enhanced control over mating (Levitan 2010).

We found within spermcasters, *F*_IS_ was significantly higher in direct developers compared to lecithotrophs and planktotrophs. In marine invertebrates, selection for risk avoidance during development (Strathmann 1985) may act on larval duration via offspring provisioning, and affect dispersal distance and inbreeding opportunities. This sets up a potential feedback loop, where opportunities for inbreeding due to short dispersal promote the purging of inbreeding depression and may then favor offspring with reduced larval dispersal. This mirrors patterns in terrestrial plants, in which species with seeds dispersed by gravity have lower genetic diversity due to inbreeding (Hamrick and Godt 1996), and supports the notion that dispersal and mating systems are linked via an evolutionary syndrome, in which species with limited dispersal are selected to inbreed and outbreeding species evolve high dispersal rates (Shields 1982; Hamrick and Godt 1996; Auld and de Casas 2013; but see Li and Pechenik 2007).

Despite similar sperm transfer, development, and sexual modes, the mating systems of different species may evolve in opposite directions due to differences in the magnitude of inbreeding depression. For instance, we found high variance in *F*_IS_ across species of gonochoristic spermcasters with direct development. At the high end, *Epiactis lisbethae*, a brooding sea anemone with crawl‐away offspring, harbored substantial homozygosity across allozyme loci with a mean *F*_IS_ of 0.95, likely reflecting restricted dispersal and biparental inbreeding (Edmands and Potts 1997). At the low end, the brooding cup coral *Balanophyllia elegans*, despite a lack of planktonic dispersal, had a mean *F*_IS_ of 0.04 (Hellberg 1994, 1996). This species either has considerable ability to discriminate against planktonic sperm of kin or suffers from severe inbreeding depression such as some terrestrial plants (Lande et al. 1994).

Given the similarities in the magnitude of *F*_IS_, factors proposed to explain the balance of inbreeding and outbreeding in plants, such as the need for reproductive assurance, the severity of inbreeding depression, and the inclusive fitness advantages of mating with kin, could help explain what drives this balance in marine invertebrates and marine macroalgae (Kokko and Ots 2006; Puurtinen 2011). Furthermore, the diversity of developmental and sperm transfer modes employed by marine organisms offers unmatched opportunities for expanding our understanding of inbreeding and outbreeding, especially for documenting the complex feedback between mating systems and the evolution of dispersal, mate choice, and hermaphroditism in marine and terrestrial organisms.

## CONFLICT OF INTEREST

The authors declare no conflict of interest.

Associate Editor: K. Monro

Handling Editor: D. W. Hall

## Supporting information


**Table S1**. Results of robust non‐parametric ANOVA for the effects of organismal group (terrestrial plants, marine macroalgae, marine invertebrates), marker type (allozyme, microsatellite) and their interaction on species level estimates of *F*_IS_.

Supplementary Material

Supplementary Material

Supplementary Material
