# Supplementary material for: Inbreeding shapes the evolution of marine invertebrates
Source: Evolution. 2020 Apr 7;74(5):871–82. doi: 10.1111/evo.13951 (PMC7383701; doi:10.1111/evo.13951)
Supplement: Supplementary file 1 — Table S1. Results of robust non‐parametric ANOVA for the effects of organismal group (terrestrial plants, marine macroalgae, marine invertebrates), marker type (allozyme, microsatellite) and their interaction on species level estimates of F IS. [file EVO-74-871-s001.docx]

**Supplementary Tables:**

Table S1. Results of robust non-parametric ANOVA for the effects of organismal group (terrestrial plants, marine macroalgae, marine invertebrates), marker type (allozyme, microsatellite) and their interaction on species level estimates of *F_IS_*. Analysis conducted separately using microsatellite estimates for 8 marine invertebrate species with *F_IS_* from both marker types (first value), and using *F_IS_* values ≥ 0 (second value). See text for details. Significant p-values in bold.

| Source | DF | RD | Mean RD | F | p-value |
| --- | --- | --- | --- | --- | --- |
| Taxon | 2 | 0.41 \| 0.14 | 0.20 \| 0.07 | 3.04 \| 1.29 | **0.049** \| 0.277 |
| Marker | 1 | 0.08 \| 0.09 | 0.08 \| 0.09 | 1.14 \| 1.69 | 0.285 \| 0.195 |
| Taxon x Marker | 2 | 0.40 \| 0.08 | 0.20 \| 0.04 | 3.02 \| 0.77 | **0.050** \| 0.465 |
