# Supplementary material for: Inbreeding shapes the evolution of marine invertebrates
Source: Evolution. 2020 Apr 7;74(5):871–82. doi: 10.1111/evo.13951 (PMC7383701; doi:10.1111/evo.13951)
Supplement: Supplementary file 2 — Supplementary Material [file EVO-74-871-s002.docx]

Entry #

1. Dupont, L. F.Viard, M.J. Dowell, S.C. Wood, and D.D. Bishop. 2009. Fine- and regional-scale genetic structure of the exotic ascidian Styela clava (Tunicata) in southwest England, 50 years after its introduction. Mol. Ecol. 18:442-453.

2. Ellis, C. D., D.J. Hodgson, C. L. Daniels, M. Collins, and A. G. F. Griffiths. 2017. Population genetic structure in European lobsters: implications for connectivity, diversity and hatchery stocking. Mar. Ecol. Prog. Ser. 563: 123 – 137.

3. Foster, N.L., C.B. Paris, J.T Kool, I.B. Baums, J.R. Stevens, J.A. Sanchez, C. Bastidas, C. Agudelo, P. Bush, O. Day, R. Ferrari, P. Gonzalez, S. Gore, R. Guppy, M.A. McCartney, C. McCoy, J. Mendez, A. Srinivasan, S. Steiner, M.J.A. Vermeij, E. Weil, and P.J. Mumby. 2012. Connectivity of Caribbean coral populations: complementary insights from empirical and modelled gene flow. Mol. Ecol. 21(5): 1143-1157.

4. Goldstein, D.R. Scheil and N.J. Gemmell. 2010. Regional connectivity and coastal expansion: differentiating pre-border and post-border vectors for the invasive tunicate *Styela clava*. Mol. Ecol. 19:874-885.

5. Guardiola, M., J. Frotscher, and M. J. Uriz. 2016. High genetic diversity, phenotypic plasticity, and invasive potential of a recently introduced calcareous spong, fast spreading across the Atlanto – Mediterranean basin. Mar. Biol. 163: 123.

6. Herborg, L.M., D. Weetman, C. Van Oosterhout, and B. Hänfling. 2007. Genetic population structure and contemporary dispersal patterns of a recent European invader, the Chinese mitten crab, *Eriocheir sinensis*. Mol. Ecol. 16(2): 231-242.

7. Jorde, P.E., G. Sovik, J-I Westgard, J. Albretsen, C. Andre, C. Hvingel, T. Johansen, A.D. Sanvik, M. Kingsley and K.E. Jorstad. 2015. Genetically distinct populations of the northern shrimp, *Pandalus borealis*, in the North Atlantic: adaptation to different temperatures as an isolating factor. Mol. Ecol. 24:1742-1757.

8. Kenchington, E.L., U. Patwary, E. Zouros, and C.J. Bird. 2006. Genetic differentiation in relation to marine landscape in a broadcast-spawning bivalve mollusk (*Placopecten magellanicus*). Mol. Ecol. 15: 1781 – 1796.

9. Kenchington, E.L., G.C. Harding, M.W. Jones, and P.A. Prodoehl. 2009. Pleistocene glaciation events shape genetic structure across the range of the American lobster, *Homarus americanus*. Mol. Ecol. 18(8): 1654-1667.

10. Kenkel, C.D., G. Goodbody-Gringley, D. Caillaud, S.W. Davies, E. Bartels and M.V. Matz. 2013. Evidence for a host role in thermotolerance divergence between populations of the mustard hill coral (*Porites astereoides*) from different reef environments. Mol. Ecol. 22:4335-4348.

11. Polato, N. R., G.T. Conception, R.J. Toonen, and I.B. Baums. 2010. Isolation by distance across the Hawaiian Archipelago in the reef-building coral *Porites lobata*. Mol. Ecol. 19: 4661 – 4677.

12. Porto-Hannes, I., A.L. Zubillaga, T.L. Shearer, C. Bastidas, C. Salazar, M.A. Coffroth, and A.M. Szmant. 2015. Population structure of the corals *Orbicella faveolata* and *Acropora palmata* in the Mesoamerican Barrier Reef System with comparisons over Caribbean basin-wide spatial scale. Mar. Biol. 162(1): 81-98.

13. Richardson, M.F., C.D.H. Sherman, R.S. Lee, N.J. Bott and A. J. Hirst. 2016. Multiple dispersal vectors drive range expansion in an invasive marine species. Mol. Ecol. 25:5001-5014.

14. Riquet, F., C. Daguin-Thiébaut, M. Ballenghien, N. Bierne, and F. Viard. 2013. Contrasting patterns of genome-wide polymorphism in the native and invasive range of the marine mollusc *Crepidula fornicata*. Mol. Ecol. 22: 1003 – 1018.

15. Roterman, C.N., J.T. Copley, K.T. Linse, P.A. Tyler, and A.D. Rogers. 2016. Connectivity in the cold: the comparative population genetics of vent‐endemic fauna in the Scotia Sea, Southern Ocean. Mol. Ecol. 25(5): 1073-1088.

16. Saarman, N.P. and G. H. Pogson. 2015. Introgression between invasive and native blue mussels (genus *Mytilus*) in central California hybrid zone. Mol. Ecol. 24:4723-4738.

17. Schiavina, M., A.M. Marino, L. Zane, and P. Melià. 2014. Matching oceanography and genetics at the basin scale. Seascape connectivity of the Mediterranean shore crab in the Adriatic Sea. Mol. Ecol. 23: 5496 – 5507.

18. Schwaninger, H.R.1999. Population structure of the widely dispersing marine bryozoan *Membranipora membranacea* (Cheilostomata): implications for population history, biogeography, and taxonomy. Mar. Biol. 135(3): 411-423.

19. Schweinsberg, M., L.C. Weiss, S. Striewski, R. Tollrian and K. P. Lampert. 2015. More than one genotype: how common is intracolonial genetic variation in scleractinian corals? Mol. Ecol. 24:2673-2685.

20. Selkoe, K. A., J. R. Watson, C. White, T. B. Horin, M. Iacchei, S. Mitarai, D. A. Siegel, S. D. Gaines, and R. J. Toonen. 2010. Taking the chaos out of genetic patchiness: seascape genetics reveals ecological and oceanographic drivers of genetic patterns in three temperate reef species. Mol. Ecol. 19: 3708 – 3726.

21. Serrano, X., I.B. Baums, K. O'reilly, T.B. Smith, R.J. Jones, T.L Shearer, F.L.D. Nunes, and A.C. Baker. 2014. Geographic differences in vertical connectivity in the Caribbean coral *Montastraea cavernosa* despite high levels of horizontal connectivity at shallow depths. Mol. Ecol. 23(17): 4226-4240.

22. Silva, C.N.S., and J.P.A. Gardner. 2015. Emerging patterns of genetic variation in the New Zealand endemic scallop Pecten novaezelandiae. Mol. Ecol. 24:5379-5393.

23. Addison, J.A., B.S. Ort, K.A. Mesa, and G.H. Pogson. 2008. Range-wide genetic homogeneity in the California sea mussel (*Mytilus californianus*): a comparison of allozymes, nuclear DNA markers, and mitochondrial DNA sequences. Mol. Ecol. 17: 4222-4232.

24. Andras, J.P., K.L. Rypien, and C.D., Harvell. 2013. Range‐wide population genetic structure of the Caribbean Sea fan coral, *Gorgonia ventalina*. Mol. Ecol. 22(1): 56-73.

25. Banks, S.C., S.D. Ling, C.R. Johnson, M. P. Piggot, J.E. Williamson and L. B. Beheregaray. 2010. Genetic structure of a recent climate change-driven range extension. Mol. Ecol. 19:2011-2024.

26. Barcia, A.R., G.E. Lopez, D. Hernandez, and E. Garcia-Machado. 2005. Temporal variation of the population structure and genetic diversity of *Farfantepenaeus notialis* assessed by allozyme loci. Mol. Ecol. 14: 2933-2942.

27. Baums, I.B., J.N. Boulay, N.R. Polato, and M.E. Hellberg. 2012. No gene flow across the Eastern Pacific Barrier in the reef‐building coral *Porites lobata*. Mol. Ecol. 21(22): 5418-5433.

28. Blanquer, A. and M.J. Uriz. 2010. Population genetics at three spatial scales of a rare sponge living in fragmented habitats. BMC Evol. Biol. 10:13.

29. Bock, D.G., A. Zhan, C. Lejeusne, H.J. MacIsaac, and M.E. Cristescu. 2011. Looking at both sides of the invasion: patterns of colonization in the violet tunicate *Botrylloides violaceus*. Mol. Ecol. 20: 503-516.

30. Boissin, E., J.P. Féral, and A. Chenuil. 2008. Defining reproductively isolated units in a cryptic and syntopic species complex using mitochondrial and nuclear markers: the brooding brittle star, *Amphipholis squamata* (Ophiuroidea). Mol. Ecol. 17(7): 1732-1744.

31. Bouchemousse, S., C. Liautard-Haag, N. Bierne and F. Viard. 2016. Distinguishing contemporary hybridization from past introgression with postgenomic ancestry-informative SNPs in strongly differentiated *Ciona* species. Mol. Ecol. 25:5527-5542.

32. Briones, C. P. Presa, M. Perez, A. Pita, and R.Guinez. 2013. Genetic connectivity of the ecosystem engineer *Perumytilus purpuratus* north to the 32°S southeast Pacific ecological discontinuity. Mar. Biol. 160: 3143-3156.

33. Brown, R.R., C.S. Davis, and S.P. Leys. 2017. Clones or clans: the genetic structure of a deep‐sea sponge, *Aphrocallistes vastus*, in unique sponge reefs of British Columbia, Canada. Mol. Ecol. 26(4): 1045-1059.

34. Sun, X. and D. Hedgecock. 2017. Temporal genetic change in North American Pacific oyster populations suggests caution in seascape genetics analysis of high gene-flow species. Mar. Ecol. Prog. Ser. 565:79-93.

35. Tepolt, C.K., and S.R. Palumbi. 2015. Transcriptome sequencing reveals both neutral and adaptive genome dynamics in a marine invader. Mol. Ecol. 24: 4145-4158.

36. Warner, P.A., M.J. van Oppen, and B.L. Willis. 2015. Unexpected cryptic species diversity in the widespread coral *Seriatopora hystrix* masks spatial‐genetic patterns of connectivity. Mol. Ecol. 24(12): 2993-3008.

37. Whalen, S., M.J. Johnson, E. Harvey and C. Battershill. 2005. Mode of reproduction, recruitment, and genetic subdivision in the brooding sponge *Haliclona* sp. Mar. Biol. 146:425-433.

38. Xiao, J., J.F. Cordes, H. Wang, X. Guo, and K.S. Reece. 2010. Population genetics of *Crassostrea ariakensis* in Asia inferred from microsatellite markers. Mar. Biol. 157: 1767-1781.

39. Yasuda, N., S. Nagai, M. Hamaguchi, K.E.N. Okaji, K. Gerard, and K. Nadaoka. 2009. Gene flow of *Acanthaster planci* (L.) in relation to ocean currents revealed by microsatellite analysis. Mol. Ecol. 18(8): 1574-1590.

40. York, K.L., M.J. Blacket and B.R. Appleton. 2008. The Bassian Isthmus and the major ocean currents of southeast Australia influence the phylogeography and population structure of a southern Australian intertidal barnacle *Catomerus polymerus* (Darwin). Mol. Ecol. 17:1948-1961.

41. Zhan, A., H.J. MacIsaac, and M.E. Cristescu. 2010. Invasion genetics of the *Ciona intestinalis* species complex: from regional endemism to global homogeneity. Mol. Ecol. 19: 4678-4694.

42. Wei, K., A.R. Wood, and J.P. Gardner. 2013. Population genetic variation in the New Zealand greenshell mussel: locus-dependent conflicting signals of weak structure and high gene flow balanced against pronounced structure and high self-recruitment. Mar. Biol. 160(4): 931-949.

43. Teske, P.R., J.Sandoval-Castillo, M. Sasaki and L.B. Behergaray. 2015. Invasion success of a habitat-forming marine invertebrate is limited by lower-than-expected dispersal ability. Mar. Ecol. Prog. Ser. 536:221-227.

44. Cahill, A.E., and J.S. Levinton. 2016. Genetic differentiation and reduced genetic diversity at the northern range edge of two species with different dispersal modes. Mol. Ecol. 25: 515-526.

45. Calderón, I., C. Palacín, and X. Turon. 2009. Microsatellite markers reveal shallow genetic differentiation between cohorts of the common sea urchin *Paracentrotus lividus* (Lamarck) in northwest Mediterranean. Mol. Ecol. 18(14): 3036-3049.

46. Carlon, D.B. and C. Lippe. 2011. Estimation of mating systems in Short and Tall ectomorphs of the coral *Favia fragum*. Mol. Ecol. 20:812-828.

47. Carrea, C., C.P. Burridge, C.K. King, and K.J. Miller. 2016. Population structure and long-term decline in three species of heart urchins *Abatus* spp. near-shore in the Vestfold Hills region, East Antarctica. Mar. Ecol. Prog. Ser. 545: 227-238.

48. Casado‐Amezùa, P., S. Goffredo, J. Templado, and A. Machordom. 2012. Genetic assessment of population structure and connectivity in the threatened Mediterranean coral *Astroides calycularis* (Scleractinia, Dendrophylliidae) at different spatial scales. Mol. Ecol. 21(15): 3671-3685.

49. Cassista M.C. and M.W. Hart. 2007. Spatial and temporal genetic homogeneity in the Artic surfclam (*Mactromeris polynyma*). Mar. Biol. 152:569-579.

50. Chaves-Fonnegra, A., K.A. Feldheim, J. Secord, and J.V. Lopez. 2015. Population structure and dispersal of the coral-excavating sponge *Cliona delitrix*. Mol. Ecol. 24: 1447-1466.

51. Costantini, F., C. Fauvelot, and M. Abbiati. 2007. Genetic structuring of the temperate gorgonian coral (*Corallium rubrum*) across the western Mediterranean Sea revealed by microsatellites and nuclear sequences. Mol. Ecol. 16(24): 5168-5182.

52. Cowart, D. A., C. Huang, S. Arnaud-Haond, S. L. Carney, C. R. Fisher and S. W. Schaeffer. 2013. Restriction to large-scale gene flow vs. regional panmixia among cold seep *Escarpia* spp. (Polychaeta, Sibloglinidae). Mol. Ecol. 22:4147-4162.

53. Dailianis, T., C.S. Tsigenoploulos, C. Dounas, and E. Voultsiadou. 2011. Genetic diversity of the imperilled bath sponge *Spongia officinalis* Linnaeus, 1759 across the Mediterranean Sea: patterns of population differentiation and implications for taxonomy and conservation. Mol. Ecol. 20: 3757-3772.

54. Dawson, M.N., R.K. Grosberg, Y.E. Stuart, and E. Sanford. 2010. Population genetic analysis of a recent range expansion: mechanisms regulating the poleward range limit in the volcano barnacle *Tetraclita rubescens*. Mol. Ecol. 19: 1585-1605.

55. Diz, A.P. and P. Presa. 2008. Regional patterns of microsatellite variation in *Mytilus galloprovincialis* from the Iberian Pennisula. Mar. Biol. 154:227-286.

56. Guiles, E.C., P. Saenz-Agudelo, N.E. Hussey, T. Ravasi, and M.L. Berumen. 2015. Exploring seascape genetics and kinship in the reef sponge *Stylissa carteri* in the Red Sea. Ecol. Evol. 5(13): 2487-2502.

57. Lal, M.M., P.C. Southgate, D.R. Jerry, C. Bosserelle, and K.R. Zenger. 2017. Swept away: ocean currents and seascape features influence genetic structure across the 18,000 Km Indo-Pacific distribution of a marine invertebrate, the black-lip pearl oyster *Pinctada* *margaritifera*. BMC Genomics 18:66.

58. Lemer, S., and S. Planes 2014. Effects of habitat fragmentation on the genetic structure and connectivity of the black-lipped pearl oyster *Pinctada margaritifera* populations in French Polynesia. Mar. Biol. 161:2035-2049.

59. Lin, Y., and A. Zhan. 2016. Population genetic structure and identification of loci under selection in the invasive tunicate, *Botryllus schlosseri*, using newly developed EST-SSRs. Biochem. Syst. Ecol. 66: 331-336.

60. Maturana, C.S., K. Gerard, A. Diaz, B. David, J. Feral, and E. Poulin. 2016. Mating system and evidence of multiple paternity in the Antarctic brooding sea urchin *Abatus agassizii*. Polar Biol. 40: 787-797.

61. McFadden, C.S. and K.Y. Aydin. 1996. Spatial autocorrelation analysis of small-scale genetic structure in a clonal soft coral with limited larval dispersal. Mar. Biol. 126:215-224.

62. Miller, K.J., and R.M. Gunasekera. 2017. A comparison of genetic connectivity in two deep sea corals to examine whether seamounts are isolated islands or stepping stones for dispersal. Sci. Rep. 7: 46103.

63. Miller, K.J., C.N. Mundy, and S. Mayfield. 2014. Molecular genetics to inform spatial management in benthic invertebrate fisheries: a case study using the Australian Greenlip Abalone. Mol. Ecol. 23: 4958-4975.

64. Mokhtar-Jamaï, K., M. Pascual, J.B. Ledoux, R. Coma, J.P. Féral, J. Garrabou, and D. Aurelle. 2011. From global to local genetic structuring in the red gorgonian *Paramuricea clavate*: the interplay between oceanographic conditions and limited larval dispersal. Mol. Ecol. 20: 3291 – 3305.

65. Nowland, S.J., P.C. Southgate, R.K. Basiita, and D.R. Jerry. 2017. Elucidation of fine-scale genetic structure of sandfish (*Holothuria scabra*) populations in Papua New Guinea and northern Australia. Mar. Freshw. Res. 68(10): 1901-1911.

66. Pemberton, A.J., L. J. Hansson, S.F. Craig, R.N. Hughes, and J.D.D. Bishop. 2007. Microscale genetic differentiation in a sessile invertebrate with cloned larvae: investigating the role of polyembryony. Mar. Biol. 153: 71-82.

67. Pérez-Portela, R., M. Rius, and A. Villamor. 2016. Lineage splitting, secondary contacts and genetic admixture of a widely distributed marine invertebrate. J. Biogeogr. 44(2): 446 – 460.

68. Pineda, M.C., X. Turon, R. Perez-Portela, and S. Lopez-Legentil. 2016. Stable populations in unstable habitats: temporal genetic structure of the introduced ascidian *Styela plicata* in North Carolina. Mar. Biol. 163: 59.

69. Aguilar, L.A., D.G. Roberts, T.E. Minchinton, and D.J. Ayre. 2015. Genetic differentiation in the barnacle *Catomerus polymerus* despite migration across a biogeographic barrier. Mar. Ecol. Prog. Ser. 524: 213-224.

70. Arnaud-Haond, S., V. Vonau, C. Rouxel, F. Bonhomme, J. Prou, E. Goyard, and P. Boudry. 2008. Genetic structure at different spatial scales in the pearl oyster (*Pinctada margaritifera cumingii*) in French Polynesian lagoons: beware of sampling strategy and genetic patchiness. Mar. Biol. 155(2) : 147 – 157.

71. Bayha, K.M., M.H. Chang, C.L. Mariani, J.L. Richardson, D.L. Edwards, T.S. DeBoer, C. Mosely, E. Aksoy, M.B. Decker, P.M. Gaffney, G.R. Harbison, J.H. McDonald, A. Caccone. 2014. Worldwide phylogeography of the invasive ctenophore *Mnemiopsis leidyi* (Ctenophora) based on nuclear and mitochondrial DNA data. Biol Invasions 17: 827-850.

72. Blanquer, A., M. Uriz, and J. Caujape-Castells. 2009. Small-scale spatial genetic structure in *Scopalina lophyropoda*, an encrusting sponge with philopatric larval dispersal and frequent fission and fusion events. Mar. Ecol. Prog. Ser. 380: 95-102.

73. Cahill, A.E., and F. Viard. 2014. Genetic structure in native and non-native populations of the direct-developing gastropod *Crepidula convexa*. Mar. Biol. 161: 2433 – 2443.

74. Calderon, I., N. Ortega, S. Duran, M. Becerro, M. Pascual, and X. Turon. 2007. Finding the relevant scale: clonality and genetic structure in a marine invertebrate (*Crambe crambe*, Porifera). Mol. Ecol. 16: 1799-1810.

75. Cardenas, L., J.C. Castilla, and F. Viard. 2016. Hierarchical analysis of the population genetic structure in *Concholepas concholepas*, a marine mollusk with a long-lived dispersive larva. Marine Ecology 37: 359-369.

76. Chen, Y., S. Li, Y. Lin, H. Li, and A. Zhan. 2017. Population genetic patterns of the solitary tunicate, *Molgula manhattensis*, in invaded Chinese coasts: large-scale homogeneity but fine scale heterogeneity. Mar. Biodiv. 48: 2137 – 2149.

77. Iacchei, M., T. Ben-Horin, K.A. Selkoe, C.E. Bird, F.J. Garcia-Rodriguez, and R.J. Toonen. 2013. Combined analyses of kinship and F_ST_ suggest potential drivers of chaotic genetic patchiness in high gene-flow populations. Mol. Ecol. 22: 3476-3494.

78. Johnson, C.H., and R.M. Woollacott. 2015. Analyses with newly developed microsatellite markers elucidate the spread dynamics of *Tricellaria inopinata* d’Hondt and Occhipinti-Ambrogi, 1985 – a recently established bryozoan along the New England seashore. Aquatic Invasions 10: 135-145.

79. Johnson, C.H. and R.M. Woollacott. 2010. Larval settlement preference maximizes genetic mixing in an inbreeding population of a simultaneous hermaphrodite (*Bugula stolonifera*, Bryozoa). Mol. Ecol. 19: 5511 – 5520.

80. Johnson, C.H., and R.M. Woollacott. 2012. Seasonal patterns of population structure in a colonial marine invertebrate (*Bugula stolonifera*, Bryozoa). Biol Bull 222: 203-213.

81. Karahan, A., J. Douek, G. Paz, and B. Rinkevich. 2016. Population genetic features for persistent, but transient *Botryllus schlosseri* (Urochordata) congregations in a central California marina. Molecular Phylogenetics and Evolution 101: 19-31.

82. Karl, S.A. and K.A. Hayes. 2012. Extreme population subdivision in the crown conch (*Melongena corona*): Historical and contemporary influences. J. Hered. 103(4): 523 – 532.

83. Teske, P.R., J. Sandoval-Castillo, M. Sasaki, and L.B. Beheregaray. 2015. Invasion success of a habitat-forming marine invertebrate is limited by lower-than-expected dispersal ability. Mar. Ecol. Prog. Ser. 536: 221-227.

84. Veliz, D., P. Duchesne, E. Bourget, and L. Bernatchez. 2006. Genetic evidence for kin aggregation in the intertidal acorn barnacle (*Semibalanus balanoides*). Mol. Ecol. 15: 4193-4202.

85. Warner, P.A., B.L. Willis, and M. J. H. Van Oppen. 2016. Sperm dispersal distances estimated by parentage analysis in a brooding scleractinian coral. Mol. Ecol. 25: 1398 – 1415.

86. Weber, A.A.T., B. Merigot, S. Valiere, and A. Chenuil. 2015. Influence of the larval phase on connectivity: strong differences in the genetic structure of brooders and broadcasters in the *Ophioderma longicauda* species complex. Mol Ecol. 24: 6080-6094.

87. Xue, D., T. Zhang, Y. Li, and J. Liu. 2017. Genetic diversity and population structure of the veined rapa whelk *Rapana venosa* along the coast of China based on microsatellites. Fish Sci 83: 563-572.

88. Puritz, J.B., C.C. Keever, J.A. Addison, S.S. Barbosa, M. Byrne, M.W. Hart, R.K. Grosberg, and R.J. Toonen. 2017. Life-history predicts past and present population connectivity in two sympatric sea stars. Ecol. Evol. 7: 3916 – 3930.

89. Reem, E., J. Douek, G. Paz, G. Katzir, and B. Rinkevich. 2017. Phylogenetics, biogeography and population genetics of the ascidian *Botryllus schlosseri* in the Mediterranean Sea and beyond. Mol. Phylogenet. Evol. 107: 221-231.

90. Riesgo, A., R. Perez-Portela, L. Pita, G. Blasco, P.M. Erwin, and S. Lopez-Legentil. 2016. Population structure and connectivity in the Mediterranean sponge *Ircinia fasciculata* are affected by mass mortalities and hybridization. Heredity 117: 427-439.

91. Rojas-Hernandez, N., D. Veliz, M. P. Riveros, J. P. Fuentes, and L. M. Pardo. 2016. Highly connected populations and temporal stability in allelic frequencies of a harvested crab from the Southern Pacific Coast. Plos one. 11(11): e0166029.

92. Tay, Y.C., M.W.P. Chng, W.W.G. Sew, F.E. Rheindt, K.P.P. Tun, and R. Meier. 2016. Beyond the coral triangle: high genetic diversity and near panmixia in Singapore’s populations of the broadcast spawning sea star *Protoreaster nodosus*. R. Soc. Open sci. 3: 160253.

93. Ledoux, J.B., J. Garrabou, O. Bianchimani, P. Drap, J.P. Feral, and D. Aurelle. 2010. Fine-scale genetic structure and inferences on population biology in the threatened Mediterranean red coral, *Corallium rubrum*. Mol. Ecol. 19: 4204-4216.

94. Lee, H. J. and E.G. Boulding. 2009. Spatial and temporal population genetic structure of four northeastern Pacific littorinind gastropods: the effect of mode of larval development on variation at one mitochondrial and two nuclear DNA markers. Mol. Ecol. 18: 2165 – 2184.

95. Levitan, D.R., N.D. Fogarty, J. Jara, K.E. Lotterhos, and N. Knowlton. 2011. Genetic, spatial, and temporal components of precise spawning synchrony in reef building corals of the *Montastraea annularis* species complex. Evolution 65(5): 1254-1270.

96. Lind, C.E., B.S. Evans, J.J.U. Taylor, and D.R. Jerry. 2007. Population genetics of a marine bivalve, *Pinctada maxima*, throughout the Indo-Australia Archipelago shows differentiation and decreased diversity at range limits. Mol. Ecol. 16: 5193-5203.

97. Macdonald, A. H. H., M. H. Schleyer, and J.M. Lamb. 2011. *Acropora austere* connectivity in the south-western Indian Ocean assessed using nuclear intron sequence data. Mar. Biol. 158: 613 – 621.

98. Matabos, M., E. Thiébaut, D. Le Guen, F. Sadosky, D. Jollivet, and F. Bonhomme. 2008. Geographic clines and stepping-stone patterns detected along the East Pacific Rise in the vetigastropod *Lepetodrilus elevatus* reflect species crypticism. Mar. Biol. 153(4): 545-563.

99. Mathews, L.M. 2007. Evidence for restricted gene flow over small spatial scales in a marine snapping shrimp *Alpheus angulosus*. Mar. Biol. 152: 645-655.

100. McKeown N. J., L. Hauser, and P. W. Shaw. 2017. Microsatellite genotyping of a brown crab *Cancer Pagurus* reveals fine scale selection and ‘non-chaotic’ genetic patchiness within a high gene flow system. Mar. Ecol. Prog. Ser. 566: 91 – 103.

101. Miller, K.J., and D.J. Ayre. 2008. Population structure is not a simple function of reproductive mode and larval type: insights from tropical corals. J. Anim. Ecol. 77(4): 713-724.

102. Miller, A.D., A. van Rooyen, G. Rasic, D.A. Ierodiaconou, H.K. Gorfine, R. Day, C. Wong, A.A. Hoffmann, and A.R. Weeks. 2016. Contrasting patterns of population connectivity between regions in a commercially important mollusc *Haliotis rubra*: integrating population genetics, genomics, and marine LiDAR data. Mol. Ecol. 25: 3845-3864.

103. Muths, D., D. Davoult, D. Gentil, and D. Jollivet. 2006. Incomplete cryptic speciation between intertidal and subtidal morphs of *Acrocnida brachiate* (Echinodermata: Ophiuroidea) in the Northeast Atlantic. Mol. Ecol. 15: 3303 – 3318.

104. Ovenden, J.R., D. Peel, R. Street, A.J. Courtney, S.D. Hoyle, S.L. Peel, and H. Podlich. 2007. The genetic effective and adult census size of an Australian population of tiger prawns (*Penaeus esculentus*). Mol. Ecol. 16(1): 127-138.
