# Supplementary figures and images for: Inbreeding shapes the evolution of marine invertebrates
Source: Evolution. 2020 Apr 7;74(5):871–82. doi: 10.1111/evo.13951 (PMC7383701; doi:10.1111/evo.13951)

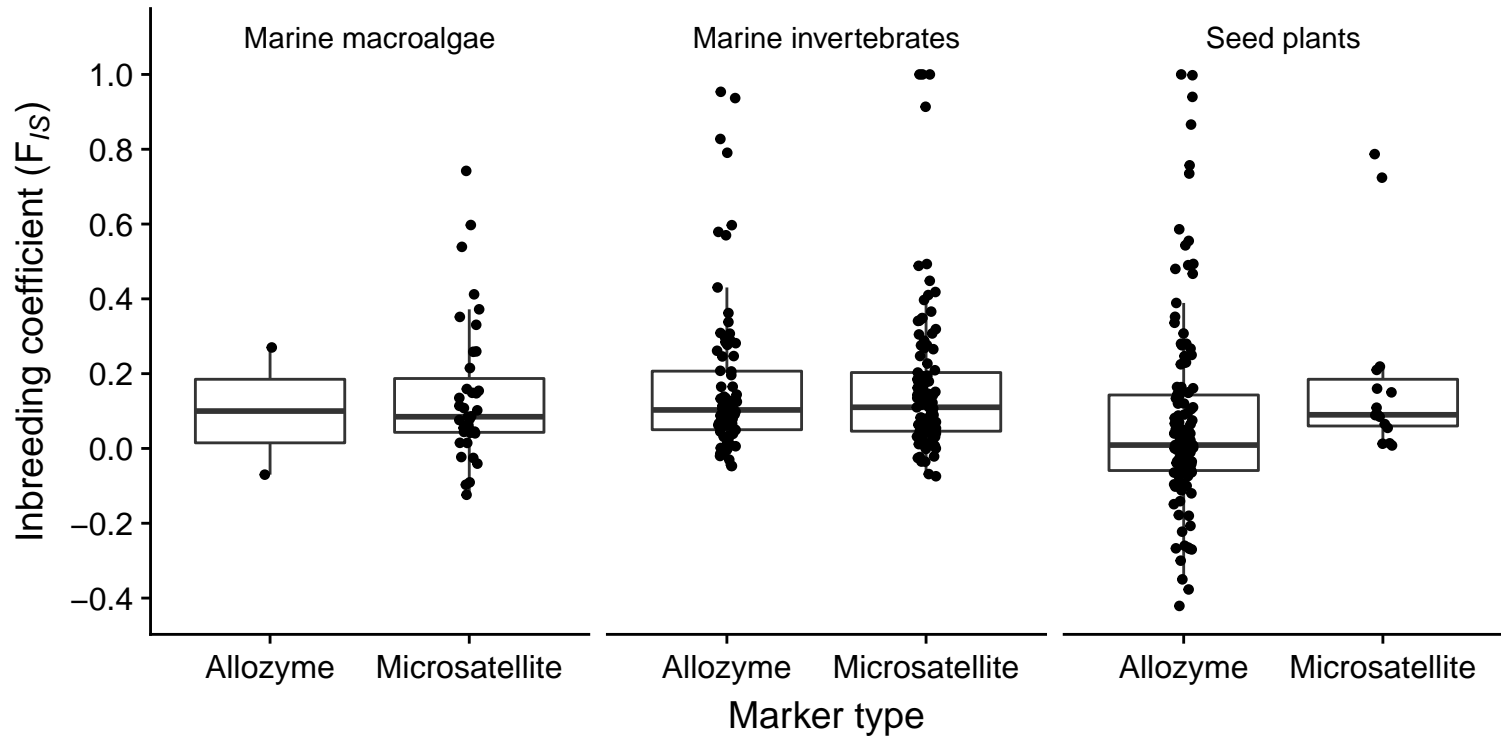

Supplement: Supplementary file 3 — Supplementary Material [file EVO-74-871-s003.pdf]

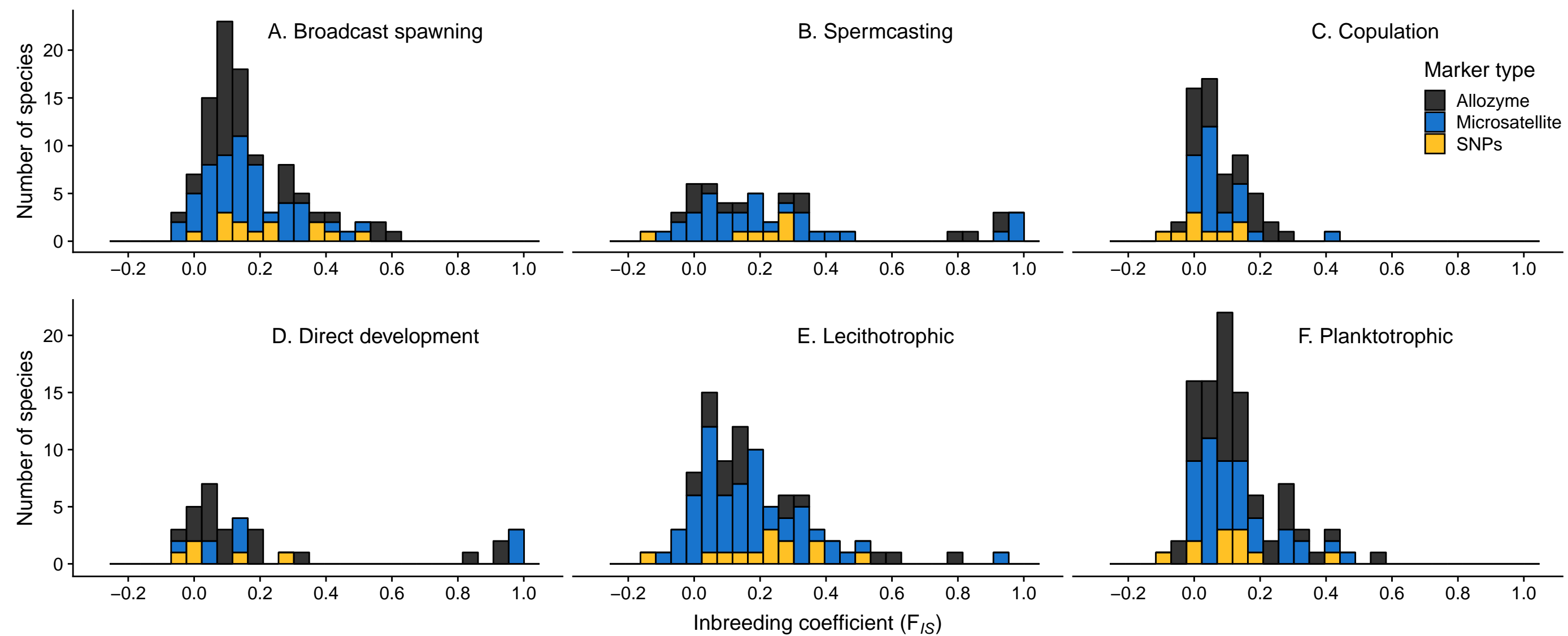

Supplement: Supplementary file 4 — Supplementary Material [file EVO-74-871-s004.pdf]
